# Relationship between Long-Term Residential Green Exposure and Individuals’ Mental Health: Moderated by Income Differences and Residential Location in Urban China

**DOI:** 10.3390/ijerph17238955

**Published:** 2020-12-01

**Authors:** Xue Zhang, Suhong Zhou, Rongping Lin, Lingling Su

**Affiliations:** 1 School of Geography and Planning, Sun Yat-sen University, Guangzhou 510275, China; zhangx396@mail2.sysu.edu.cn (X.Z.); linrp3@mail2.sysu.edu.cn (R.L.); sulling@mail2.sysu.edu.cn (L.S.); 2 Guangdong Provincial Engineering Research Center for Public Security and Disaster, Guangzhou 510275, China

**Keywords:** green exposure, mental health, residential location, China, Guangzhou

## Abstract

Environmental health effects during urbanization have attracted much attention. However, knowledge is lacking on the relationship between long-term cumulative residential environment and health effects on individuals during rapid transformations in urban physical and social space. Taking Guangzhou, China, as a case example, this study analyzed the relationship between long-term exposure to green environments and residents’ mental health under urban spatial restructuring. Based on a household survey in 2016, 820 residents who have lived in Guangzhou for more than 15 years were used as the sample. High-resolution remote sensing images were used to assess the long-term green exposure of residents. The results indicate that long-term green exposure in residential areas had a negative correlation with residents’ mental health (*p* < 0.05), and the correlation was strongest for the cumulative green environment in the last five years. However, this significant effect was moderated by income and residential location. Green exposure had a positive relationship with mental health for low income groups, and a negative relationship for middle and high income groups. In addition, residents living farther away from the city center were likely to have fewer green environmental health benefits. Residential relocation in a rapidly urbanizing and transforming China has led to the continuous differentiation of residential green environments among different income groups, which has also caused different mental health effects from green exposure. It provides empirical evidence and theoretical support for policymakers to improve the urban environment and reduce environmental health disparities by considering social differences and residential location.

## 1. Introduction

After four decades of institutional transformation in China, rapid urban social and economic development has triggered an increasing risk of mental disorders. Mental and neurological diseases ranked first in the total burden of diseases in China, accounting for about 20% of the total burden of diseases in recent years [1]. People are becoming increasingly aware that psychological disorders are affected by both individual characteristics and environmental exposure, and the influence of residential location on health exceeds the factors of individual level [2,3,4,5,6]. Residential relocation connects urban space and individual behavior, and determines the environmental context of individuals, which is considered the source and persistence of environmental health differences [7,8]. Thus, more attention should be paid to the effect of residential environmental change during institutional transitions on the mental health of individuals.

The effects of social and environmental factors on mental health have been widely demonstrated [5,9,10]. There are three hypotheses on their association: psychosocial stress theory; concentrated disadvantage, and social mobility [9]. Research has shown that mental health problems are associated with living environments [11]. People living in poor communities are often at higher risk of mental disorders because of the disadvantages of environment, infrastructure, and housing [8]. In addition, the homogeneity of neighborhoods in poor communities also results in a disadvantaged social environment, such as neighborhood poverty, higher social crime, smoking, drinking, and other unhealthy behaviors. The psychosocial stress theory suggests that these negative circumstances can generate stress, psychological vulnerability, and depression, which affect mental health [12]. Socioeconomic status is associated with environmental hazards and various characteristics of housing and neighborhood environments [13]. The explanation of concentrated disadvantage indicates that low income groups are at a concentrated disadvantage in housing conditions, environmental exposure, neighborhood environment, social support, public services, and health care, which all intensify the risk of mental disorders [14]. Social migration interpretation proposes that, to some extent, residential relocation is the major cause of continuous population differentiation in environmental health [8]. In areas vulnerable to environmental hazards, the affluent gradually move away from the adverse living environments to improve their mental health, while low-income groups are unable to improve their living conditions and eventually suffer from both environmental hazards and mental diseases.

In addition to the inequality of exposure to environmental hazards, the ability to access resources for health benefits such as green space is increasingly considered as part of environmental justice [15]. Healthy cities are required to provide a range of natural and built environments to support and promote health, recreation, and wellbeing [16]. Green space is considered to be an important environmental component to improve mental health and has attracted wide attention. It can alleviate the attention fatigue caused by prolonged mental effort in daily life and enhance the quality of mindfulness and happiness (Attention Restoration Theory, ART) [17]. However, few studies have included the green environment in the framework integrating psychosocial theory and environmental health. Particularly in the context of China’s social and economic reform, it is important to understand the environmental health effects of China’s transformation on society, cities, and residents, by studying the relationship between urban residential relocation, the green environment, and mental health.

The short-term health effects of green exposure have been documented in numerous studies [18,19,20]. Increasingly, recent studies have focused on the mental health effects of long-term green exposure to provide evidence about causality for epidemiological studies on green environments and health [21,22,23,24,25]. Exposure to green environments and the resulting mental health outcomes are correlated with factors of social and spatial dimensions such as individual attributes and living space [21,26,27,28]. It is necessary to understand the effects of green environments on mental health from multiple dimensions of time, space, and society, and understand the complex interaction between them. This paper aims to explore the relationship between long-term green exposure and individual mental health under China’s residential relocation policies. The following three questions are discussed. First, what is the relationship between long-term green exposure in residential environments with residents’ mental health? Second, from the social dimensions, does the relationship vary according to individual attributes? Last, from the spatial dimensions, does the relationship vary according to residential location?

Section 2 of the paper explores the relationship between institutional reform, residential relocation, and social class stratification, as well as group differences in environmental health. The analytical framework of the empirical research is proposed through a literature review on the relationship between green environments and mental health. Section 3 introduces the study design. Section 4 reports the result of the empirical research. Section 5 presents the empirical research conclusions and discussion, and the corresponding policy implications.

## 2. Analytical Framework and Literature Review

### 2.1. Relationship between Institutional Reform, Residential Relocation, and Social Class Stratification

A substantial increase in residential relocation was driven from the transformation of China’s social and economic development. During the period of the planned economy, China’s urban housing supply was mainly dependent on state-owned enterprises and units. As a type of welfare, housing was provided to each employee in the form of unit housing, which resulted a “unit system” housing pattern [29,30,31]. The abolition of the welfare housing distribution policy in 1998 was an important turning point. After housing marketization, a large number of residents sought housing from the market, which led to large-scale intraurban residential relocation.

The social stratification and housing inequality caused by the institutional transformation and housing reform have increased, and the economic status of urban residents has been differentiated. On the one hand, the market transition led to a large number of industrial workers in state-owned enterprises being excluded from the newly established labor market and trapped in a new urban poor class [30,32]. On the other hand, during the transition period, the unequal distribution of housing benefits within units and the horizontal inequity between units rapidly widened the income gap between different groups [29,31,33]. Benefit clusters in society were reconstructed because of profitability. The profit or loss change process led to social differentiation [34]. With increasing marketization, housing as a consumer product has been rank-developed as affordable housing, ordinary commercial housing, high-grade residences, and so on, is used to meet the needs of different income levels. Constrained by income level, demand, and individual preferences, the beneficiaries continued to have better housing conditions, while vulnerable groups lacked the ability to improve and therefore remain in a housing dilemma. The pattern of housing inequality based on income levels is constantly being consolidated and deepened [31,35].

### 2.2. Differences in Environmental Health Effects by Income Group

Under China’s rapid transformation of society and the economy, residents’ living conditions and environment have persistent differences due to residential relocation and class differentiation, which has severe effects on individuals’ mental health. The exposure–disease–stress model for environmental health disparities explains the relationship between ethnicity, environment, and health conditions [8]. It points out that there is a high correlation between race and residential location; however, different living locations lead to different health risks and outcome. In China, residential segregation is based on residents of different socioeconomic attributes and not race, reflected in the continuous differentiation of individual socioeconomic status and neighborhood context and social environment [36]. Low income groups tend to be more exposed to environmental hazards, including hazardous waste, environmental and indoor air pollutants, water pollution, environmental noise, and crowded housing [37,38], and their health is adversely affected [39,40]. In addition to environmental hazard inequality in residential areas, the ability to access beneficial resources is also increasingly considered by environmental justice research and the health benefits of exposure to green environments in residential areas are widely recognized.

### 2.3. Effects of Green Environment on Mental Health

There are several accepted explanations for the effects of green environment on mental health: (1) a green environment can alleviate the mental fatigue caused by excessive concentration in daily life and enhance the quality of mindfulness and happiness (Attention Restoration Theory, ART) [17], (2) a green environment can increase people’s physical activity to promote health [41,42], (3) green public spaces are conductive to social interaction [43], and (4) a green environment often indicates a better health environment where there is less air pollution and noise [44,45]. There is abundant evidence on the beneficial effects of exposure to green environments on mental health [23,46,47,48,49,50,51].

However, the mental health impacts of green space exposure reflected in multiple temporal, spatial, and social dimensions are conditioned by complicated interactions. From the temporal dimensions, most studies based on experimental assessments have shown the beneficial effects of short-term green exposure. For example, nature exposure promoted positive changes in attention, memory, and mood in youth according to systematic review of 59,221 papers [52]. In addition, long-term exposure to green may improve residents’ behavior and lead to cumulative health outcome. Evidence shows that long-term experiences with the natural environment facilitated social connection [18,41,42,43] and encouraged physical activity [53,54], which was thus beneficial to mental health. In addition, some medical research studies have shown that health problems are partly the cumulative effect of long-term environmental exposure [55,56]. Long-term exposure to living in a green environment can reduce psychological disorders such as anxiety and depression [57]. People exposed to more greenery in childhood have improved mental health in adulthood [58] and reduced risk of psychiatric disorders [25,59].

In the spatial dimension, the distribution location, type, and quality of green space may lead to different mental health outcomes. On the one hand, uneven distribution of environmental space can cause an unequal burden and benefit of living environments [26], such as the most affluent areas have a higher proportion of greenery than the least affluent in Australia’s most populous cities [21]. On the other hand, the research in China shows that there are large differences between urban and suburban residents in the use of green space and physical exercise due to the availability and quality of green space [60,61], which may result in different mental health outcomes over time.

In the social dimension, individual attributes such as gender, age, education, income, health, and health adverse behaviors may have strong moderating effects on the relationship between green exposure and mental health [26,27,28]. The main reason is individual differences in perceptions and use of urban green space, as well as further behavioral differences due to the impact of social background, class, and residential area. For example, in Sweden, greenery provides more benefits for men than women, and for adults who are physically active than those physically inactive [62].

Besides, there are still other uncertainties in the relationship between green environment and mental health that may affect the final outcome of the relationship, such as the assessment of the green environment [23], where common measurement indicators include greenness, proportion of green area, and accessibility of green space, assessment of green quality, and individual perceptual or visible green. The normalized difference vegetation index (NDVI) is often used to measure green environment or exposure to the surrounding greenery in many studies [48,59].

In western countries, some scholars have focused on the long-term effects of green environments on mental health, but little is known in China. In particular, in the context of China’s institutional transformation and increasing intraurban residential relocation, social class differentiation and housing differentiation have led to prominent income-based environmental inequality [63]. Based on the logical relationships between the social transformation context, residential relocation, environmental exposure, and long-term health outcomes, it is important to investigate the relationship of long-term residential green exposure and mental health under residential relocation in China’s transformation period.

### 2.4. Conceptual Framework and Research Hypothesis

**Figure 1:**
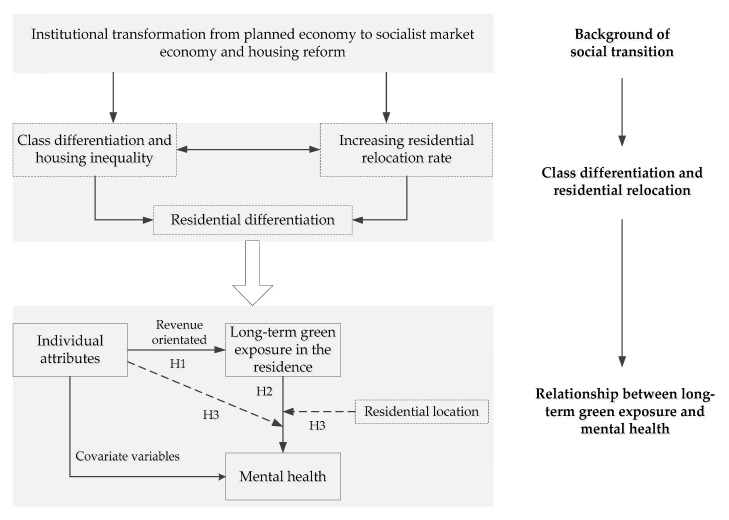
Conceptual framework. Note: H1 = Hypothesis 1. H2 = Hypothesis 2. H3 = Hypothesis 3.

Based on the analysis and review above, this paper proposes a conceptual framework to examine and compare the relationship of long-term green exposure and mental health under residential relocation (Figure 1). There are three hypotheses.

**Hypothesis** **1.** *The current mental health of residents is related to the long-term residential green exposure.*

**Hypothesis** **2.** *This relationship may be moderated by social factors such as individual attributes and residents’ behaviors.*

**Hypothesis** **3.** *This relationship may also be moderated by spatial factors such as residential location.*

## 3. Study Design

### 3.1. Survey Participants

This study used data from a cross-sectional survey of intraurban residence relocation and public health conducted in Guangzhou, China, in 2016. The study area and the questionnaire survey were introduced in Zhang et al. [64]. A total of 1029 questionnaires were obtained. To investigate the relationship of longitudinal residential green exposure and mental health, a total of 820 residents who have lived in Guangzhou for 15 years or more were selected for this study. Although the 820 residents selected have not moved out of Guangzhou in the past 15 years, they have experienced intraurban residence relocation.

### 3.2. Mental Health Data

Mental health is defined as a state of emotional and psychological wellbeing. The World Health Organization’s Five Well-Being Index (WHO-5) is one of the most universal and reliable methods used for subjective mental health assessment [65,66,67]. WHO-5 is based on five questions about feeling “cheerful and in good spirits,”, “calm and relaxed”, “active and vigorous”, “fresh and rested”, and “daily life has been filled with things that interest me” during the past two weeks. Participants answer each question on a five-point scale from 0 (none of the time) to 5 (all of the time), and total scores on the WHO-5 range from 0 to 25.

### 3.3. Exposure Assessment of Residential Green Environment

Green environment in Guangzhou was assessed using Landsat 7–8 satellite images to calculate the normalized difference vegetation index (NDVI) at 30 m × 30 m spatial resolution. Because Guangzhou is located on the subtropical coast and has a maritime subtropical monsoon climate, the vegetation is always green in all seasons, with little seasonal change. Therefore, seasonal greenery differences were ignored when selecting a Landsat satellite remote sensing image for each study year. Previous results also support the stability of NDVI spatial comparisons in terms of seasons and years [45,68]. To obtain residential green exposure data for the past 15 years, cloud-free or the least cloudy images from 2001 to 2015 were used. The value of the NDVI is between −1 and +1, and a higher value means a greater density of green vegetation [69]. Negative values for NDVI were removed as they mean the ground is covered by cloud, water, etc.

**Figure 2:**
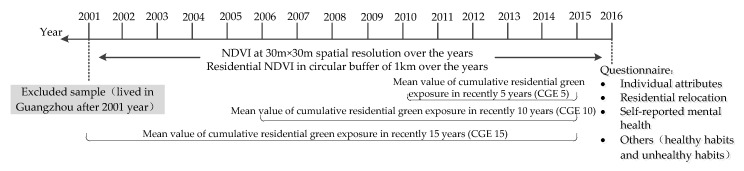
Summary of assessment and collection of variables. Note: NDVI= Normalized difference vegetation index.

Assessment of green exposure was based on residential location over the past 15 years. The questionnaire recorded the specific relocation information, including the moving time and detailed address of each move. We collected the residential location of each participant in each of the past 15 years. According to the residential location and green environment of each year, the average value of NDVI in a circular buffer of 500 m and 1 km from each survey participant’s residence was calculated according to previous research experience [64,70,71]. Cumulative residential green exposure (CGE) was calculated by taking the mean value of green exposure in the recent few years (Figure 2). We used the CGE1, CGE2, … …, CGE15 respectively to represent the mean value of green exposure in the recent 1 years, 2 years, ……, 15 years.

### 3.4. Description of Variables

Previous studies have shown that the social and economic attributes of respondents, their physical exercise, and their daily habits, as well as the geographical environment of the survey areas, have an important impact on the relationship between green environment and mental health [72]. The covariates in this study included individual attributes (socioeconomic attributes and behaviors) and residential location data, all collected in the survey.

- Individual socioeconomic attributes

Individual socioeconomic attributes include gender, age, education level, and personal monthly income. As long-term green exposure of residents at different income levels may have different effects on mental health, according to Statistics Bureau of Guangzhou Municipality, the per capita disposable income of urban residents was 4245 Yuan in 2016, participants were divided into three levels: low income group of <3000 Yuan (10.0%), middle income group of 3000–6999 Yuan (69.1%), and high income group >7000 Yuan (20.9%).

- Individual behaviors

Healthy habits were recorded. Physical exercise has been proven to be beneficial to physical and mental health. Using the “part 4: recreation, sport, and leisure-time physical activity” of the last seven days self-administered version of the international physical activity questionnaire [73], we collected the frequency and duration of three levels of physical activity over the past seven days: “brisk walking (for recreational and transportation purposes)”, “moderate physical activities (dancing, playing bowling/ping-pong/badminton, and so on)”, and “vigorous physical activities (aerobic exercise, running, fast cycling, swimming, playing basketball/football, and so on)”. Based on the International Physical Activity Questionnaire Analysis Guide [74], metabolic equivalents (METs) are commonly used to measure the intensity of physical activities and for the analysis of data. The METs for brisk walking and moderate and vigorous physical activities are 3.3, 4.0, and 8.0 MET, respectively. To represent the weekly physical activity intensity of each resident, the METs equal to physical activity frequency × duration × MET for the three types of physical activity were calculated and summed.

Habits known to be adverse for health were also recorded. Excessive drinking and smoking are common harmful behaviors. The history of drinking (at least once a week for half a year or more) and smoking (at least one cigarette per week for one year or more) were recorded in the questionnaire. The questions on respondents yes or no to smoking or drinking were also used in this study.

For neighborhood communication, the survey asked “the number of residents (adults) in the community who greet each other when you meet them, except family members and relatives”, with answers including “below 10 people”, “10 to 20 people”, “21 to 30 people”, “31 to 50 people”, and “more than 50 people”, valued 1 to 5, respectively. The more people who say hello, the more closely the neighborhood relationship.

- Residential location

Distance from the city center is an important indicator of geographical location, which also reflects the residential preference of different residents. Under the mode of urban centripetal development in China, living closer to the city center means denser road networks and public service facilities, as well as more employment opportunities and shorter commuting distance, which may affect the mental health of residents [71]. However, the geographical locations to some extent also represent different living conditions. The residential space differentiation in Guangzhou can be divided into three categories: the old residential area, the “unit” residential area, and the new residential area from the downtown to the outskirts. The geometric distance between each respondent’s residence and the city center (Beijing road traditional center and Zhujiang New Town center; city centers were selected from the overall 2011–2020 planning of Guangzhou city) was calculated as the location index, and the shortest geometric distance was taken to represent the geographical space location of a respondent’s residence.

### 3.5. Methods

Since the dependent variable of mental health measured using WHO-5 was a continuous quantitative variable from 0 to 25, a linear regression model was used to study the association between long-term cumulative residential green exposure and mental health at the individual level. The analysis included several steps. First, a descriptive statistical analysis was performed. Then, multiple linear regression models (main effect model) were conducted to explore the relationship between mental health and long-term residential green exposure (residential green exposure in 2015 (CGE1), in 2011–2015 (CGE5), in 2006–2015 (CGE10), and in 2001–2015 (CGE15), model 1a–1d), controlling the indicators of residents’ attributes and residential location. Last, the interaction terms between moderating variables and the measure of green environment (normalized difference vegetation index, NDVI) were separately added in turn to models 1a–1d to examine the moderating effects of gender (model 2a–2d), age (model 3a–3d), educational levels (model 4a–4d), income (model 5a–5d), healthy behaviors (model 6a–6d), behaviors adverse to health (including history of smoking and drinking, model 7a–7d and model 8a–8d), neighborhood interaction (model 9a–9d), and residential location (model 10a–10d) on long-term residential green exposure and mental health. The interaction models were specified as follows:

(1) $${\mathrm{MH}}_{i}=\alpha +\beta_{1}Gender_{i}+\beta_{2}Age_{i}+\beta_{3}\mathrm{Education}\;{\mathrm{level}}_{i}+\beta_{4}{\mathrm{Income}}_{i}+\beta_{5}{\mathrm{Smoking}}_{i}+\;\beta_{6}{\mathrm{Drinking}}_{i}+\beta_{7}{\mathrm{PE}}_{i}+\beta_{8}\mathrm{Neighborhood}\;{\mathrm{interaction}}_{i}+\beta_{9}\mathrm{Residential}\;{\mathrm{location}}_{i}+\;\beta_{10}NDVI\_CGE_{iy}+\beta_{11}NDVI\_CGE_{iy}\times Regulator_{i}+\varepsilon_{iy}$$

where *i* represents individual *i*; α is the intercept; and $\beta_{1}$ to $\beta_{10}$ represent the regression coefficient of gender, age, education level, income, smoking, drinking, physical exercise, neighborhood interaction, residential location, and cumulative green exposure, respectively. $NDVI\_CGE_{iy}$ represents cumulative residential green exposure of the last *y* years of individual *i* (e.g., when *y* = 2, it means the last 2 years, that is, years 2014 and 2015); $Regulator_{i}$ represent variables of individual attributes and residential location; $\beta_{11}$ represents the coefficient of interactions; and $\varepsilon_{iy}$ represents random error terms. The significance test of the interaction coefficient (*p* < 0.05) indicated that the covariates have a moderating effect.

In order to reduce the bias caused by the assessment buffer of green exposure, we calculated all models by using the value of NDVI in a circular buffer of 500 m and 1 km, and found that the range of assessment buffer has no obvious influence on the results. Only the results based on the value of NDVI in a circular buffer of 1 km are listed and analyzed in this paper.

## 4. Results

### 4.1. Study Sample Characteristics

The mean age of the study participants was 42.7 years (SD 13.67), half were female, and 46.6% had achieved technical secondary school or higher education degree, and nearly half of them had monthly personal incomes below 5000 Yuan. In terms of health behaviors, 40.85% had a history of smoking and 26.59% had a history of drinking, the average weekly physical activity intensity of each resident was 12.41 METs, and nearly 75% of the sample say hello to no more than 20 neighbors in their community (Table 1).

### 4.2. Association of Long-Term Residential Green Exposure and Mental Health

Table 2 shows the results of multiple linear regression. Model 1a–1d estimated the relationship between mental health and green exposure of residents in 2015 (CGE1), in 2011–2015 (CGE5), in 2006–2015 (CGE10), and in 2001–2015 (CGE15), where model 1a was a reference. The index of age, physical exercise, distance to city center, and NDVI were standardized. The results indicated an inverse association between cumulative green exposure in residents and mental health, and all of the associations reached statistical significance. For instance, in model 1d for exposure over 2001–2015, the coefficient of cumulative green exposure in the last 15 years was −0.75 (95% CI: −0.99, −0.51) for mental health. Hypothesis 1 that current mental health status is related to the long-term residential green exposure has been verified. However, the mechanism is complicated.

**Figure 3:**
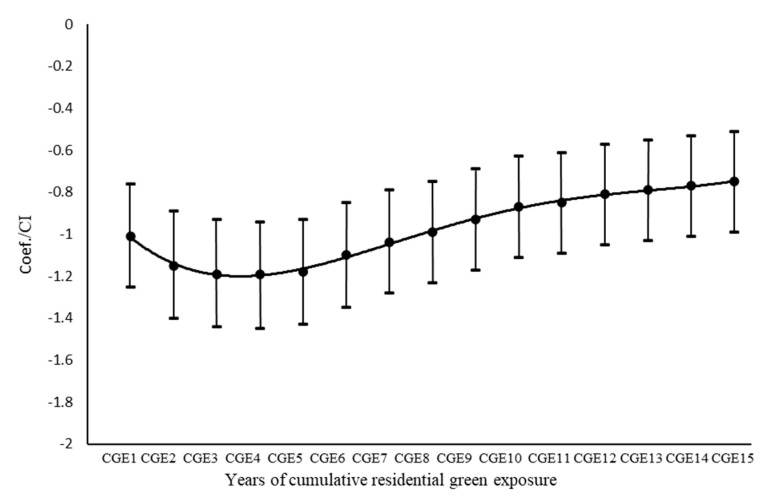
Association of cumulative green exposure (CGE) and mental health. Note: Coef. = coefficient. CI = 95% confidence interval. CGE1, CGE2, …, CGE15 respectively represent the mean value of green exposure in the recent 1 year, 2 years, …, 15 years.

Cumulative green exposure in the last two years, the last three years, and up to the last 14 years were calculated according to the same method. The correlation coefficients of NDVI and mental health were also computed to better compare the association between green environment on mental health from different cumulative times (Figure 3). The variables of gender, age, education level, history of smoking, history of drinking, physical exercise, and distance to city center were controlled (all coefficients *p* ≤ 0.05 and variance inflation factors (VIF) < 5). Results showed that the relationship between mental health and the green exposure in the last five years was strongest (Figure 3).

Moreover, residents’ mental health was related to individual attributes and residential location. Education level, income, physical exercise, and neighborhood communication were all significantly positively correlated with mental health, while age, history of smoking, and distance to city center were significantly negatively correlated.

### 4.3. Mediators of Individual Attributes and Residential Location

Table 3 shows the moderating effect of individual attributes and residential location on the association between cumulative residential green exposure and mental health. The models in the first to fourth columns respectively represent the results of the green exposure of residents in 2015 (CGE1), in 2011–2015 (CGE5), in 2006–2015 (CGE10), and in 2001–2015 (CGE15), where green exposure in 2015 (CGE1) is the reference. The gender, age, education, income, smoking, drinking, physical exercise, neighborhood communication, and residential location were controlled in all models. Only the coefficients of NDVI and interaction terms in the model are listed in Table 3.

No statistical significant modification was found for gender (model 2a–2d), age (model 3a–3d), education level (model 4a–4d), history of smoking (model 6a–6d), history of drinking (model 7a–7d), and physical exercise (model 9a–9d) on the relationship between greening exposure and mental health, regardless of the cumulative green exposure in the past 1, 5, 10, or 15 years (Table 3, interaction coefficient *p* > 0.05). There was a significant, positive interaction of lower income and high income for the relationship between cumulative green exposure and mental health, respectively, in model 5a–5d and model 5c–5d, where middle income was the reference. A significantly positive interaction was found for neighborhood communication on the association between mental health and cumulative green exposure in the past 5 years. For the association between mental health and cumulative green exposure in the past 10 and 15 years, a significantly negative interaction was found for distance to city center.

Thus, only income and residential location obviously moderated the relationship between green exposure and mental health, confirming Hypotheses 2 and 3.

- Moderating effect of income

The interaction between income and green exposure could account for 0.7% to 1% of the variance in mental health in model 5a–5d, in the analysis of the cumulative green exposure in the past 1, 5, 10, and 15 years, respectively. The results of model 5a–5d also showed that the NDVI coefficient values were −1.21, −1.44, −1.05, and −0.94, respectively, which showed there was a significant negative association between green exposure and mental health in the middle income group. The coefficient of income (low) × NDVI referred to the difference in mental health effects of long-term green exposure between the low income group and the middle income group when the level of greenery changed. For example, an increase of one unit in greenery resulted in a health effect of −1.21 for middle income group and 0.43 for low income group, a difference of 1.64. Similarly, the coefficient of income (high) × NDVI referred to the difference in mental health effects of long-term green exposure between the high income group and the middle income group.

**Figure 4:**
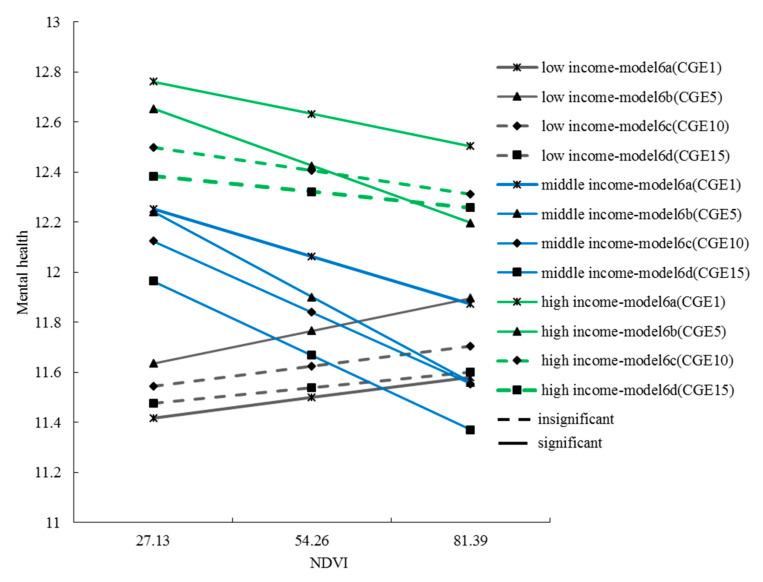
The relationship between long-term green exposure and mental health under three income levels. Note: NDVI =Normalized difference vegetation index. CGE1, CGE5, CGE10, CGE15 respectively represent the mean value of green exposure in the recent 1 year, 5 years, 10 years, and 15 years.

According to the income difference of the effect of green exposure on mental health, a relationship diagram was plotted to represent the long-term green exposure effect on mental health under three income levels (Figure 4). The relatively recent residential green environment (within the past five years) had a significant positive correlation with the mental health of the low income group, and a significant negative correlation with the high income group, and also a negative correlation for the middle income group in all models.

- Moderating effect of residential location

**Figure 5:**
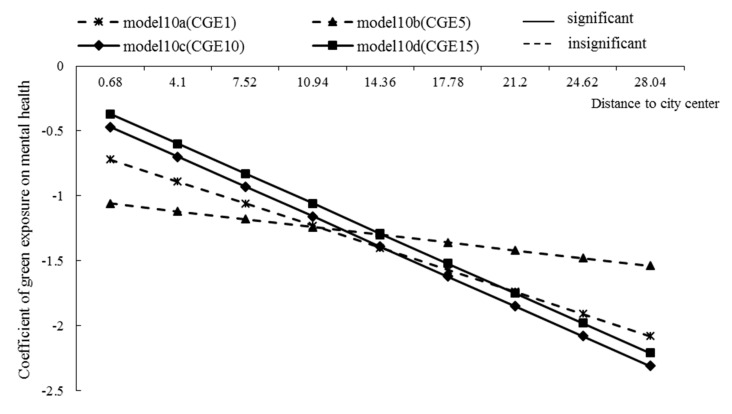
The moderating effect of distance to city center on the relationship between long-term green exposure and mental health. CGE1, CGE5, CGE10, CGE15 respectively represent the mean value of green exposure in the recent 1 year, 5 years, 10 years, and 15 years.

The results of model 11a–d showed that the coefficients of distance to city center × NDVI were −0.17, −0.05, −0.24, and −0.24, respectively, which represented the slope of the effect of green exposure on mental health, which decreased by 0.17, 0.05, 0.24, and 0.24 units, respectively, for each unit increase in the distance from a residence to the city center. However, the moderating effects were only significant in models 11c and 11d (Figure 5). This meant that the farther away the residence was from the city center, the greater the negative effect of the surrounding green environment on mental health, especially over a longer period of time.

## 5. Discussion

The extensive Chinese economic reform has greatly improved general population health; however, the rapid development of urbanization has also brought new challenges to public health. Social class stratification, residential differentiation, and rapid changes in the urban environment have led to increasingly prominent environmental and health inequalities related to poverty [75]. At the individual level, institutional reform benefited the majority of residents while also causing social, environmental, and health disadvantages for vulnerable groups, which requires much research and policy attention. This paper discussed the relationship of long-term residential green exposure and individuals’ mental health during the transition period of economic and social reform. Using 15 years of green exposure data from Guangzhou, the study found that there is significant correlation of long-term green exposure and residents’ mental health. The relationship of residential green exposure on mental health also varies due to the cumulative time, residents’ income, and residential location, which shows multiple interactions in the dimensions of time, society, and space.

### 5.1. Negative Correlation between Cumulative Green Exposure and Mental Health

This research found that long-term residential green exposure had a significant negative relationship with residents’ mental health. The cumulative effect of residential green exposure is time-limited, with the more recent the exposure to a residential green environment, the stronger the correlation with current mental health.

In previous studies, the general conclusion was that the greener the environment, the better the mental health [22,76]. Although some studies showed that there is no valid evidence for a direct relationship between residential green space and mental health, a few studies also showed a negative correlation between amount of greenery and mental health [77]. It is clear that the differences across various studies in the study area, objectives, methods, and other factors considered such as the urban environment, geographical location, built environment, and accessibility to green space may lead to inconsistent and incomparable results. There are still many uncertainties in the relationship between green environment and mental health that may affect the final outcome of the relationship.

### 5.2. Moderating Effects of Income Levels

**Figure 6:**
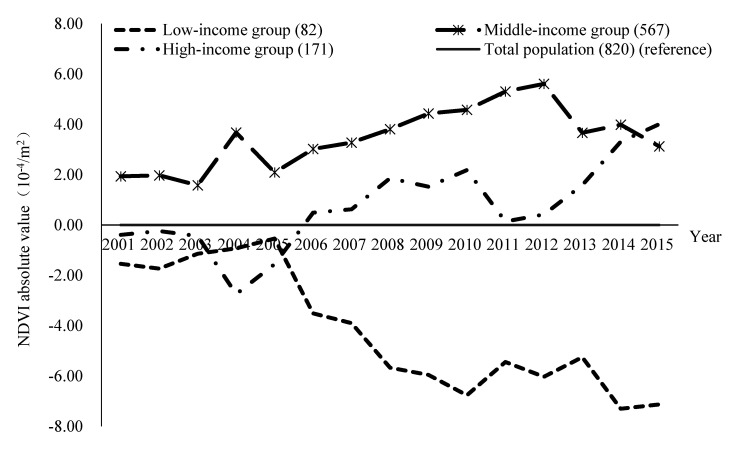
The absolute value of residential green exposure for different income groups in the past 15 years. Note: the absolute value of NDVI (Normalized difference vegetation index) for the low income group is equal to the mean NDVI of the low income group minus the mean NDVI of total population, and so on.

Environmental justice and health equity are important topics in environmental and health research. Research showed that vulnerable groups are often disadvantaged in access to environmental resources [78,79]. Two studies in the UK also concluded that high levels of green environment reduced the pressure on people living in poor socioeconomic areas [80,81]. This study in Guangzhou found some similarities with the results of previous research that the low income group had less greenery in their living environment [82,83], and greenery was positively correlated with mental health. However, as shown in Figure 6, middle income residents and high income residents lived in the greenest areas, but this had a disadvantageous effect on mental health (Figure 4). The reason may be the mediating effect of the quality and the availability of green space, which is not explored in this study. This finding also contradicts the idea that the more green exposure, the better the mental health—possibly because overgrown or poorly managed vegetation may limit people’s visibility in public spaces and make them feel insecure [84].

From the perspective of the moderating effect of income, improving the green environment in the residential areas of low income groups may be an effective measure to reduce health inequality, but the reality is that the disparity of residential green space between low income, middle income, and high income groups was increasing rapidly after 2005 (Figure 6). With further progress in urbanization and marketization, exposure to a green environment in residential areas was more unequally distributed among the general population. Low income groups are facing a shortage of the green environments that are particularly important for their mental health, but their needs are increasingly not satisfied.

### 5.3. Moderating Effects of Residential Location

In this study, the geographical location of residence was included in the model as a control and moderating variable to study the impact of spatial characteristics of green space on mental health. The results showed that the index of distance to city center was negatively correlated with mental health. For instance, the distance to city center in the last one year odds ratio for mental health is −1.06 (95% CI: −1.31, −0.81) in Table 2, and the negative moderating effect in Figure 5, which suggests that residents living farther away from the city center were likely to have fewer green environmental health benefits. This followed a previous study in Guangzhou city that found that suburban residents generally had lower mental health than inner city residents [71] because people who live far from the city center tend to spend more time on commuting. For example, in Guangzhou, the average distance from the residence to the city center is 3.01 km for the low income group, 4.40 km for the middle income group, and 4.12 km for the high income group, and the commuting distance for workers is 4.55, 5.77, and 4.97 km, respectively (calculated as the straight-line distance between the workplace and residence in 2015). Another possibility is that city dwellers were more likely to prefer manicured parks to a wilderness environment [85,86]; however, green space in the suburbs may be poorly managed in China’s cities, which may have a negative impact on mental health. Meta-analysis of previous research also found that the location of a study significantly moderated the influence of green environment on human mood [87,88].

### 5.4. Income, Residential Location, and Green Environment Health Effects

**Figure 7:**
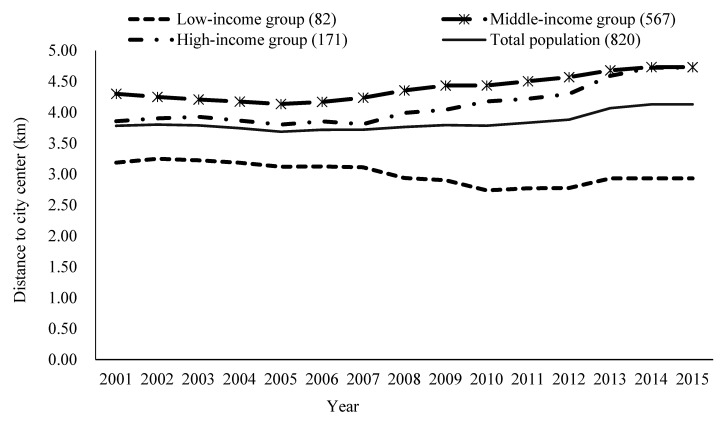
Average distance from residence to city center for residents in three income groups from 2001 to 2015.

Variation of residence location of residents in different income groups over the years is analyzed in Figure 7. The low income group generally lived closer to the city center area, while the middle income group lived farther away. This result is also consistent with the residential space differentiation in Guangzhou, which can be divided into three categories: the old residential area, the “work unit” residential area, and the new residential area from the downtown to the outskirts. Correlation analysis shows there is a significant positive correlation between the distance from residence to the city center and green environment. There is less greenery in the city center and more greenery farther away. Although the frequency of moves among the different groups is roughly the same, the average number of residential relocations is 2 for the low income group, 1.9 for the middle income group, and 1.96 for the high income group. Low income residents have had a shortage of a green environment in the central district due to their limited capability to relocate to a better environment, but a green environment has an important effect on their mental health. By moving to the outskirts of the city, residents with middle and high incomes can get more greening environment, but the health effects of greenery is no longer obvious.

## 6. Conclusions

### 6.1. Main Finding

Taking Guangzhou, China, as an example, this research examined the relationship of long-term exposure to a green residential environment and mental health in the context of residential relocation during China’s transition period. This study showed that the current mental health is related the long-term residential green exposure. The moderating effects of individual attributes and residential location on the relationship between long-term green exposure and mental health were studied. There are three main conclusions. First, long-term green exposure in the residential area had a negative correlation with residents’ mental health (*p* < 0.05), and the correlation was strongest for the cumulative green environment in the last five years. Second, there was a significant difference in the mental health effect of long-term green exposure among different income groups. The green exposure of residential areas had a positive correlation with mental health for the low income group but a negative correlation with middle income and high income groups. It reveals the deepening environmental injustice in cities during China’s institutional transition process. Low income residents are at a disadvantage in a green environment that is beneficial to their mental health; however, the distribution inequality of green environments is gradually worsening and they are trapped in areas with less greenery. Last, residential location has a regulating effect on the relationship between long-term green exposure and mental health. The farther a residence was away from the city center, the more adverse the effects of the surrounding green environment on mental health. Overall, the relocation of income groups has led to differences in environmental and health effects. With limited ability to move, low income groups have been living in lower greenery areas in the city center, facing the dilemma that a green environment is important for mental health but is increasingly scarce. The middle and high income groups are moving away from the city center and getting a richer green environment, but there are no mental health benefits and even negative effects.

### 6.2. Implications and Limitations

This paper makes a meaningful theoretical contribution to bring the urban green environment into a framework integrating psychosocial and environmental concepts [9]. It is the first to focus on the relationship between intraurban residential relocation, a long-term green environment, and mental health in the context of social and economic reform. It is vital to understand the environmental and health effects of policy changes in China on society, cities, and residents. The results of this research provide new evidence and practical implications for policy makers and planners in reducing environmental and health inequalities and building healthy cities.

In the present study, more recent the exposure to a residential green environment had stronger correlation with current mental health. This implies that residents should consider more about the potential environmental impact of their choice of residence. From the perspective of policy makers, it is important to carefully consider how the moderating effects of different income groups and urban area on green and mental health will affect the appropriate policy formulation. It may be important to improve low-density green areas in the city centers, such as adding some green belts or planning public green spaces within walking distance. However, it is also necessary to take into account the possible increase in rents and house prices caused by the improvement of green environment, which may force low-income groups with limited rent capacity to move out. In addition, improving the accessibility of urban public green space is an important way to overcome the heterogeneity of green environment between different regions and communities.

However, the study also has some limitations. First, we can only assess the long-term green environment in residential areas by remote sensing images of satellite data, which may be different from the actual observed or accessible green environments of residents. We calculated residential greenery based on historical residence location recalled by respondents over 15 years, which may suffer from memory bias and omissions that lead to inaccurate results. Second, the satellite data selected did not represent the same time every year because the images with the least clouds were used to assess the residential green environment. However, the geographical location and climate of Guangzhou means that the trees are always green, and the stability of NDVI spatial contrast across seasons and years has been demonstrated in previous studies [45,68]. We still need to be cautious when comparing the longitudinal results of NDVI values over the years as there may be seasonal differences and cloud occlusion of image data. Therefore, we used a relative comparison between the green environment values of the residential locations of three different income levels and the overall average value for the sample when analyzing green environment changes over 15 years. Finally, the assessment of individual attributes and self-reporting mental health was cross-sectional. Although most studies have used the same method [23,57], there is heterogeneity of characteristic variables at different times, which also has an effect on the results of the research. The results can only detect the correlation between long-term green exposure in the residential location and mental health in combination with other variables, but cannot show the causal relationship. Further exploration is needed on the influence mechanism of long-term exposure to green environments on mental health.

## Figures and Tables

**Table 1:** Descriptive statistics of all variables (*N* = 820).

Variable			%/Mean
Mental health (mean of WHO-5)			12.04
Individual attributes			
Individual socioeconomic attributes	Gender	Male	49.88%
		Female	50.12%
	Age (years)	19–29	21.46%
		30–44	36.95%
		45–60	26.10%
		>60	15.49%
	Education level	Senior high school or lower	53.41%
		Technical secondary school/bachelor degree	46.10%
		Master degree or higher	0.49%
	Personal monthly income (Yuan)	0–2999	10.01%
		3000–6999	69.14%
		>7000	20.86%
Individual behavior	History of smoking	Yes	40.85%
	History of drinking	Yes	26.59%
	Physical exercise	METs (/h)	12.41
	The number of residents (adults) greeted within the community	Below 10 people	29.39%
		10 to 20 people	45.85%
		21 to 30 people	16.34%
		31 to 50 people	5.73%
		More than 50 people	2.68%
Residential location	Distance to city center (km)		6.64

Note: WHO-5 = The World Health Organization’s Five Well-Being Index.

**Table 2:** Association between cumulative residential green exposure and mental health in the past 1, 5, 10, and 15 years: linear regression model (main effect model).

Variables	Model 1a (ref. CGE1)	Model 1b (CGE5)	Model 1c (CGE10)	Model 1d (CGE15)
	Coef. (CI)	Coef. (CI)	Coef. (CI)	Coef. (CI)
Gender (female = 0)	0.98 ** (0.27, 1.68)	1.00 ** (0.3,1.7)	0.97 ** (0.26, 1.68)	0.96 ** (0.24, 1.68)
Age	−0.47 *** (−0.74, −0.19)	−0.46 *** (−0.73, −0.19)	−0.42 ** (−0.7, −0.14)	−0.41 ** (−0.69, −0.13)
Education level	0.76 ** (0.23, 1.3)	0.75 ** (0.21, 1.28)	0.72 * (0.17, 1.26)	0.71 * (0.16, 1.26)
Income	0.58 * (0.14, 1.02)	0.50 * (0.07, 0.94)	0.55 * (0.1, 0.99)	0.53 * (0.08, 0.98)
History of smoking (none = 0)	−1.38 *** (−2.12, −0.64)	−1.35 *** (−2.08, −0.61)	−1.35 *** (−2.1, −0.59)	−1.33 *** (−2.08, −0.57)
History of drinking (none = 0)	−0.53 (−1.15, 0.09)	−0.58 (−1.2, 0.03)	−0.58 (−1.21, 0.05)	−0.56 (−1.2, 0.07)
Physical exercise	0.59 *** (0.36, 0.82)	0.58 *** (0.36, 0.81)	0.49 *** (0.26, 0.72)	0.45 ** (0.22, 0.68)
Neighborhood communication	0.37 ** (0.13, 0.61)	0.45 *** (0.21, 0.69)	0.42 *** (0.18, 0.66)	0.39 *** (0.15, 0.64)
Distance to city center	−1.06 *** (−1.31, −0.81)	−0.96 *** (−1.2, −0.71)	−1.2 *** (−1.44, −0.96)	−1.28 *** (−1.52, −1.05)
**NDVI**	−1.01 *** (−1.25, −0.76)	−1.18 *** (−1.43, −0.93)	−0.87 *** (−1.11, −0.63)	−0.75 *** (−0.99, −0.51)

Note: * *p* ≤ 0.05; ** *p* < 0.01; *** *p* < 0.001. ref.= reference. Coef. = coefficient. CI = 95% confidence interval. CGE1, CGE5, CGE10, CGE15 respectively represent the mean value of green exposure in the recent 1 years, 5 years, 10 years, 15 years. The index of age, physical exercise, distance to city center, and NDVI (Normalized difference vegetation index) were standardized. Variance inflation factors is less than 5.

**Table 3:** Moderating effect of individual attributes and residential location on association between cumulative residential green exposure and mental health in the past 1, 5, 10, and 15 years: interaction model.

	Model 2a (ref. CGE1)	Model 2b (CGE5)	Model 2c (CGE10)	Model 2d (CGE15)
	Coef./CI	Coef./CI	Coef./CI	Coef./CI
NDVI	−1.09 *** (−1.42, −0.75)	−1.22 *** (−1.56, −0.88)	−0.84 *** (−1.17, −0.52)	−0.66 *** (−0.98, −0.24)
Gender(male) × NDVI	0.07 (−0.28, 0.52)	0.07 (−0.27, 0.52)	−0.07 (−0.52, 0.29)	−0.19 (−0.65, 0.27)
	Model 3a (ref. CGE1)	Model 3b (CGE5)	Model 3c (CGE10)	Model 3d (CGE15)
NDVI	−1.08 *** (−1.33, −0.82)	−1.18 *** (−1.33, −0.93)	−0.87 *** (−1.11, −0.63)	−0.75 *** (−0.98, −0.51)
Age × NDVI	−0.23 (−0.37, 0.01)	−0.22 (−0.36, 0.03)	−0.18 (−0.31, 0.06)	−0.19 (−0.32, 0.03)
	Model 4a (ref. CGE1)	Model 4b (CGE5)	Model 4c (CGE10)	Model 4d (CGE15)
NDVI	−1.24 *** (−1.49, −0.90)	−1.44 *** (−1.67, −1.01)	−1.01 *** (−1.44, −0.70)	−0.89 *** (−1.21, −0.47)
Education (Technical secondary school/ bachelor degree or higher) × NDVI	0.47 (−0.09, 0.84)	0.44 (−0.12, 0.78)	0.41 (−0.14, 0.77)	0.41 (−0.14, 0.77)
	Model 5a (ref. CGE1)	Model 5b (CGE5)	Model 5c (CGE10)	Model 5d (CGE15)
NDVI (ref. middle income)	−1.21 *** (−1.49, −0.94)	−1.44 *** (−1.50, −1.04)	−1.05 *** (−1.44, −0.79)	−0.94 *** (−1.22, −0.58)
Income(low) × NDVI	1.74 ** (0.44, 4.01)	1.84 ** (0.44, 4.14)	1.45 * (0.28, 2.44)	1.14 * (0.18, 2.12)
Income(high) × NDVI	0.49 (−0.17, 0.94)	0.44 (−0.14, 1.01)	0.71 * (0.11, 1.41)	0.74 * (0.14, 1.45)
	Model 6a (ref. CGE1)	Model 6b (CGE5)	Model 6c (CGE10)	Model 6d (CGE15)
NDVI	−1.09 *** (−1.40, −0.77)	−1.21 *** (−1.42, −0.90)	−0.87 *** (−1.17, −0.47)	−0.74 *** (−1.04, −0.44)
History of smoking(1) × NDVI	0.07 (−0.49, 0.44)	0.07 (−0.49, 0.44)	−0.00 (−0.47, 0.47)	−0.02 (−0.49, 0.45)
	Model 7a (ref. CGE1)	Model 7b (CGE5)	Model 7c (CGE10)	Model 7d (CGE15)
NDVI	−1.19 *** (−1.49, −0.90)	−1.40 *** (−1.49, −1.02)	−0.94 *** (−1.21, −0.55)	−0.75 *** (−1.04, −0.49)
History of drinking(1) × NDVI	0.44 (−0.04, 0.94)	0.44 (−0.04, 0.94)	0.24 (−0.27, 0.77)	−0.05 (−0.47, 0.50)
	Model 8a (ref. CGE1)	Model 8b (CGE5)	Model 8c (CGE10)	Model 8d (CGE15)
NDVI	−0.79 *** (−1.25, −0.42)	−0.94 *** (−1.27, −0.47)	−0.74 *** (−1.07, −0.44)	−0.54 *** (−0.97, −0.44)
Number of people say hello × NDVI	0.17 (−0.11, 0.47)	0.29 * (0.01, 0.47)	0.14 (−0.11, 0.41)	0.12 (−0.14, 0.47)
	Model 9a (ref. CGE1)	Model 9b (CGE5)	Model 9c (CGE10)	Model 9d (CGE15)
NDVI	−1.04 *** (−1.41, −0.70)	−1.17 *** (−1.42, −0.92)	−0.77 *** (−1.12, −0.54)	−0.74 *** (−0.99, −0.41)
Physical exercise × NDVI	−0.17 (−0.41, 0.07)	−0.14 (−0.47, 0.07)	−0.07 (−0.44, 0.20)	−0.01 (−0.27, 0.27)
	Model 10a (ref. CGE1)	Model 10b (CGE5)	Model 10c (CGE10)	Model 10d (CGE15)
NDVI	−0.79 *** (−1.24, −0.44)	−1.12 *** (−1.44, −0.70)	−0.70 *** (−0.99, −0.42)	−0.50 *** (−0.77, −0.44)
Distance to city center × NDVI	−0.17 (−0.49, 0.04)	−0.05 (−0.2, 0.14)	−0.24 * (−0.42, −0.04)	−0.24 * (−0.42, −0.04)

Note: * *p* ≤ 0.05; ** *p* < 0.01; *** *p* < 0.001. Coef. = coefficient. CI = 95% confidence interval. Variance inflation factors (VIF < 4). NDVI =Normalized difference vegetation index. CGE1, CGE5, CGE10, CGE15 respectively represent the mean value of green exposure in the recent 1 year, 5 years, 10 years, and 15 years. The index of age, physical exercise, centrality, and NDVI were standardized. All models controlled for gender, age, education, income, smoking, drinking, physical exercise, neighborhood communication, and residential location. Only the coefficients of NDVI and interaction terms in the interaction model are listed.
